# MES7 Modulates Seed Germination via Regulating Salicylic Acid Content in Arabidopsis

**DOI:** 10.3390/plants10050903

**Published:** 2021-04-30

**Authors:** Wenrui Gao, Yan Liu, Juan Huang, Yaqiu Chen, Chen Chen, Lu Lu, Hongwei Zhao, Shuzhen Men, Xiaoming Zhang

**Affiliations:** 1 Department of Plant Biology and Ecology, College of Life Sciences, Nankai University and Tianjin Key Laboratory of Protein Science, Tianjin 300071, China; gaowenrui2020@126.com; 2 State Key Laboratory of Integrated Management of Pest Insects and Rodents, Institute of Zoology, Chinese Academy of Sciences, Beijing 100101, China; liuyan8882@126.com (Y.L.); huangjuan@ioz.ac.cn (J.H.); 15670533706@163.com (Y.C.); chenvaequ0609@163.com (C.C.); lulubio2018@163.com (L.L.); hzhao@njau.edu.cn (H.Z.); 3 CAS Center for Excellence in Biotic Interactions, University of Chinese Academy of Sciences, Beijing 100049, China; 4 Department of Life Sciences, Henan Normal University, Xinxiang 453007, China; 5 College of Plant Protection, Nanjing Agricultural University, Nanjing 210095, China

**Keywords:** seed germination, salicylic acid, methyl salicylate, *MES7*, abiotic stress

## Abstract

Seed germination is an important phase transitional period of angiosperm plants during which seeds are highly sensitive to different environmental conditions. Although seed germination is under the regulation of salicylic acid (SA) and other hormones, the molecular mechanism underlying these regulations remains mysterious. In this study, we determined the expression of SA methyl esterase (*MES*) family genes during seed germination. We found that *MES7* expression decreases significantly in imbibed seeds, and the dysfunction of *MES7* decreases SA content. Furthermore, *MES7* reduces and promotes seed germination under normal and salt stress conditions, respectively. The application of SA restores the seed germination deficiencies of *mes7* mutants under different conditions. Taking together, our observations uncover a MeSA hydrolytic enzyme, MES7, regulates seed germination via altering SA titer under normal and abiotic stress conditions.

## 1. Introduction

Plant growth and stress response are two important factors that decide the final yield of crops [1]. Meanwhile, plant growth is dramatically altered by different stresses. Plant hormones are organic substances that are synthesized by plants and play important roles in the regulation of almost all plant developmental processes. A wide variety of versatile phytohormones, including auxins, gibberellins (GA), cytokinins, brassinosteroids, salicylic acid (SA), jasmonates (JA), ethylene (ET) and abscisic acid (ABA) have been identified in plants and involves in plant development and responses to environmental stresses. SA is well known as an important signal transduction molecule that regulates plants resistance to viruses, fungi and bacteria under various biotic stresses [2,3,4]. With the deepening of research, the role of SA further extends to regulate various plant development processes, including plant growth, development, maturation, senescence and the like [5].

Seed germination (from seed to seedling) is a major developmental phase transitional period in angiosperm plants. Many growth hormones, such as ABA [6,7,8], GA [9,10,11], and ET [12], have been extensively studied in seed germination. Moreover, seeds are sensitive to environmental conditions (soil water content, oxygen and temperature). When the conditions are favorable, seeds will break its dormancy and produce radicle, the process of seed germination is completed [13]. Abiotic stresses also affect the germination of seeds, and hormones fine-turn plant responses to these stresses to achieve efficient germinations. Direct or indirect evidence suggests that SA is involved in the regulation of seed germination [14,15]. Under normal growth conditions, exogenous SA application inhibits seed germination in a dosage-dependent manner [14,15,16]. However, SA promotes seed germination under abiotic stress. Exogenous application of SA can partially reverse the inhibitory effect of oxidative and heat stress on seed germination [17]. SA also plays a positive role in seed germination by alleviating oxidative damage caused by high salt stress [14,18]. Although the functions of SA in plant defense against biotic stresses have been well studied, the precise role and the underlying molecular mechanisms of SA upon seed germination have not been fully elucidated.

SA regulation is a complicated process, which controlled by both synthesis and storage steps. Plants synthesize SA via the isochorismate (IC) and phenylalanine ammonia-lyase (PAL) enzymatic pathways [19,20]. Upon the biotic stresses, most of functional SA is produced from PAL generation pathway. For storage, most of plant SA is converted to inactive SA-glucoside (SAG) and salicyloyl-glucose (SGE) [21,22]. Methyl salicylate (MeSA) is another inactive form of SA that is synthesized from SA by SA carboxyl methyltransferase (SAMT) [23]. In the systemic tissues, inactive MeSA is converted to active SA by methyl esterase (MES) family protein and performed its roles in plant development [24,25]. Twenty members of MES have been identified in A. thaliana, which encode proteins with high sequence similarity with tobacco salicylic acid-binding protein 2 (SABP2) [26,27]. MES gene family participated in the hydrolysis of MeSA, MeJA, or MeIAA [26]. Although the crystal structure [28], hydrolytic activity [26] and SAR signaling [27,29] of MESs have been partially studied, the function of MESs on seed germination has not been determined.

We focused on whether the MES gene family is involved in the regulation of seed germination, and if so, which gene(s) play a role and what is the regulation mechanism? In light of the key role of MESs in SA content alternation and function in plant resistance to pathogens, we studied the expression of MES family genes at different germination conditions and discovered that the transcript level of MES7 dramatically response to germination condition. Two mes7 mutant lines were obtained, and the accumulation of SA decreased in the mes7 mutants, which is consistent with the function of MES7 in MeSA esterase activity [26,27]. Moreover, the mes7 mutants germinated faster under normal growth condition. The germination of mes7 mutant plants is more resistant to SA treatment than that of control plants. In contrast, the germination rate of mes7 mutant plants under high salinity is lower than that of control plants. Taken together, this study shows that MES7 plays an important role in the regulation of SA content and seed germination.

## 2. Results

### 2.1. MES7 Accumulation Deceases in Imbibed Seeds

**Figure 1:**
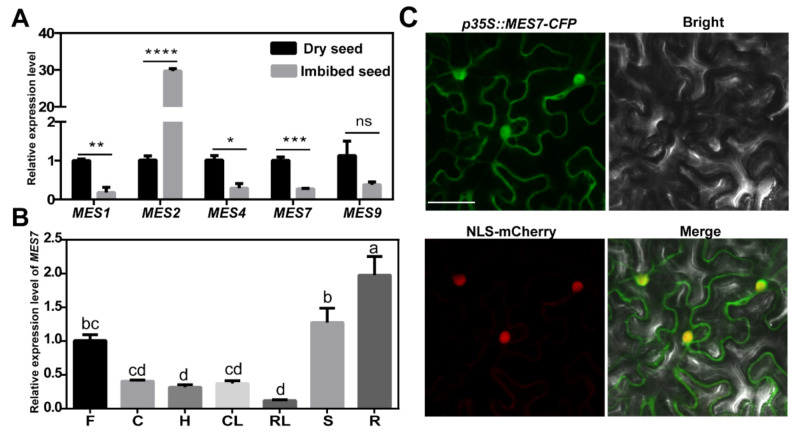
Expression and subcellular localization analysis of *MESs*. (**A**) Relative expression of *MES1*, *MES2*, *MES4*, *MES7*, and *MES9* in seeds. Col-0 wild-type (WT) seeds were imbibed on moistened filter paper for 24 h, whereas dry seeds were maintained on dry filter paper for 24 h. Total RNA was extracted from the seeds, and *GAPDH* was used as an internal control. The expression level of the gene in dry seeds was set as 1. Error bars represent standard deviation of three replicates. Asterisks mark significant differences between the dry seeds and imbibed seeds according to Student’s *t*-test, * *p*-value < 0.05, ** *p*-value < 0.01, *** *p*-value < 0.001, **** *p*-value < 0.0001, and ns means no significant difference. (**B**) Relative expression of *MES7* in different tissues of *A*. *thaliana*. R: root, S: stem, F: flower, CL: cauline leaf, RL: rosette leaf, C: cotyledon, H: hypocotyl. Cotyledons and hypocotyls were taken from one-week-old seedlings, and other tissues were taken from eight-week-old flowering *A*. *thaliana*. *ACTIN2* was used as an internal control. The expression of *MES7* in flower was set as 1. Error bars represent standard deviation of three replicates. Different lowercase letters represent significant differences of *MES7* among different tissues (*p* < 0.05) according to Tukey’s test. Primers are listed in Supplemental Table S1. (**C**) Microscopy analysis of the subcellular localization of MES7 in epidermal cells of *N. benthamiana*. Green indicates CFP. Red indicates NLS-mCherry. Scale bar, 50 μm.

MESs play essential roles in the catalyzed reaction of MeSA to SA, and AtMES1, −2, −4, −7, and −9 displayed MeSA hydrolase activity in vitro [26,27]. To determine which gene(s) play essential role in seed germination, we first detected gene abundance of *MESs* in a SA-sensitive seed growth condition. Previous study showed that genes related to SA accumulation are suppressed during the imbibition process before visible seed germination [30]. We excluded *MES2* due to its significant increase in imbibed seeds. As shown in Figure 1A, the expression levels of *MES1*, *−4*, *−7* and *−9* were all down-regulated in the imbibed seeds, and *MES7* exhibits the most significant decrease, which suggested *MES7* may involve in the SA-regulated seed germination process.

To further characterize MES7 activity in *A*. *thaliana*, we determined the expression of *MES7* in different tissues. The result showed that *MES7* lowly accumulated in rosette leaves and hypocotyls while highly accumulated in roots (Figure 1B). The subcellular localization of MES7 was further determined. As shown in Figure 1C, MES7 widely distribute in cytoplasm and nucleus.

### 2.2. Identification of MES7 Mutants

**Figure 2:**
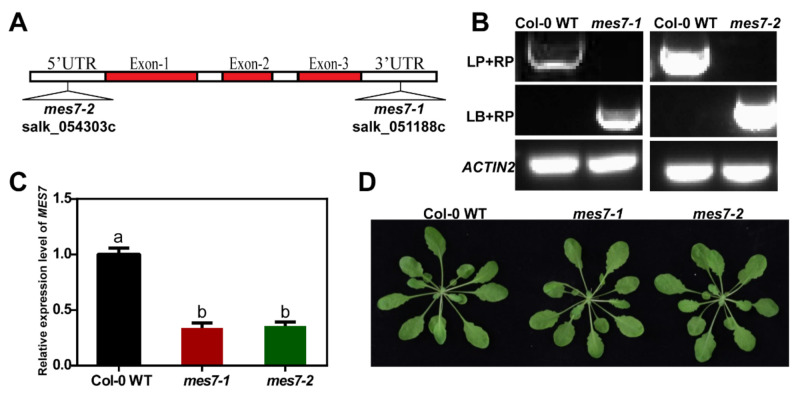
Identification of *mes7* mutants. (**A**) Scheme of *MES7* T-DNA insertion position of the two mutants: *mes7-1* (Salk_051188c), *mes7-2* (Salk_054303c). (**B**) Genotyping on *mes7-1* and *mes7-2* mutants. *ACTIN2* was used as an internal reference gene. (**C**) Relative expression level of *MES7* in mutants was analyzed by qRT-PCR. The expression of *MES7* in Col-0 WT was set as 1. *ACTIN2* was used as an internal control. Error bars represent standard error of mean of three replicates. Different lowercase letters represent significant differences of *MES7* among Col-0 WT and *mes7* mutant plants (*p* < 0.05) according to Tukey’s test. Primers are listed in Supplemental Table S1. (**D**) Phenotype of five-week-old Col-0 WT and *mes7* mutant plants.

To determine the function of MES7, two T-DNA insertion lines, *mes7-1* (Salk_051188c) and *mes7-2* (Salk_054303c), were obtained from Arabidopsis Biological Resource Center (ABRC). The mutant lines were then verified by genotyping and qRT-PCR analysis (Figure 2A–C). The accumulation of *MES7* decreased 66% and 64.33% respectively in the two *mes7* mutant lines. However, compared to Col-0 WT, *mes7-1* and *mes7-2* did not display obvious development defects (Figure 2D), which indicated that MES7 may not play significant roles in plant growth processes.

### 2.3. Dysregulation of MES7 Affects Seed Germination under Normal Condition

**Figure 3:**
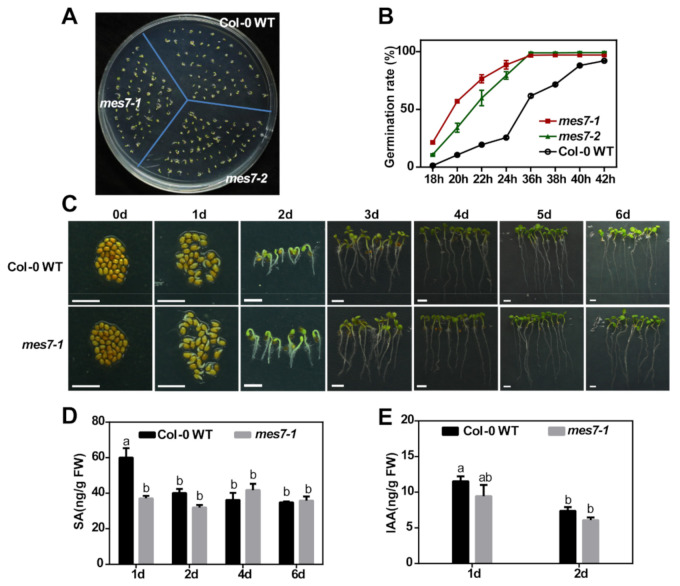
MES7 modulates seed germination under normal condition. (**A**) Germination phenotype of Col-0 WT, *mes7-1* and *mes7-2* seeds on 1/2 MS medium. Representative images were photographed at 2 day after incubation under normal germination condition. (**B**) Time course quantification of germination on 1/2 MS medium. Three biological repeats were performed for each assay and about 50 seeds were used for each repeat. Error bars represent standard error of mean of three replicates. (**C**) Phenotype of seeds and seedlings at different germination time. Scale bar, 0.2 cm. (**D**) SA content in Col-0 WT and *mes7-1*. (**E**) IAA content in Col-0 WT and *mes7-1*. Error bars indicate the standard error of the mean. Similar results were obtained for four biological replicates. For (**D**,**E**), different lowercase letters represent significant differences of SA or IAA among different samples (*p* < 0.05) according to Tukey’s test.

As the application of exogenous SA inhibits seed germination under normal condition [14,15,16] and *MES7* transcript significantly decreases during seed germination (Figure 1A), *MES7* may also modulate seed germination. With the T-DNA insertion lines isolated, we detect the function of MES7 in seed germination process. The function of MES7 in seed germination under normal growth conditions was firstly determined. Under normal growth conditions, *mes7* mutant seeds indeed germinated faster than those of Col-0 WT seeds (Figure 3A,B). When the mutants had almost two complete cotyledons, only a few of the wild type had cotyledons (Figure 3A). Germination rate of *mes7* mutants and Col-0 WT revealed that almost all seeds of *mes7* mutant germinated by 36 h, while only about 61.75% of Col-0 WT germinated (Figure 3B). These results indicate that *MES7* plays important roles in seed germination under normal growth condition.

We further determined the mechanism underlying the function of MES7 in seed germination. Previous studies showed that MES7 hydrolyze MeSA [26,27,28]. Therefore, it is possible that MES7 mediates seed germination by regulating SA accumulation. To test this possibility, the SA content during seed germination was determined. At the early stage of germination (1 day), *mes7* mutants displayed most significant seed development and SA content difference comparing to Col-0 WT (Figure 3C,D). MES7 can also cause hydrolysis of MeIAA [27]. In order to verify whether the early germination phenotype of *mes7* seeds was only due to the change of SA content rather than IAA, we measured the content of endogenous IAA. As shown in Figure 3E, there was no significant difference in the accumulation of IAA between *mes7* mutant and Col-0 WT at the early stage of germination. These results suggest that *MES7* may regulate seed germination by modulating the content of SA.

### 2.4. Exogenous SA Application Rescues the Germination Differences in MES7 Deficient Plants

**Figure 4:**
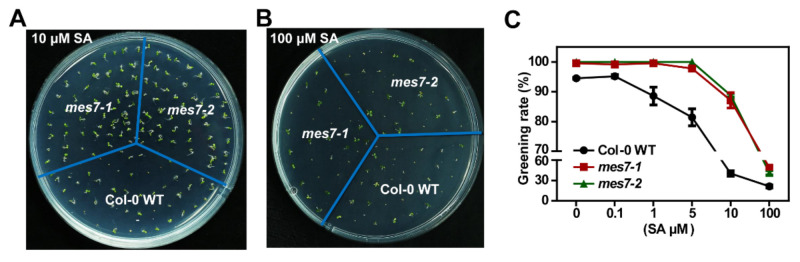
MES7 modulates seed germination in a SA-dependent manner under normal conditions. Representative image of Col-0 WT and *mes7* mutants under 10 μM SA (**A**) and 100 μM SA (**B**) treatments. (**C**) Greening index (two cotyledons exposed) of Col-0 WT and *mes7* mutant seeds under different concentrations of SA. Data collected after 3 days grow at 22 °C in the incubator. Data are mean ± SEM of three replicates and about 40 seeds were used for each repeat.

To further explore the correlation between MES7 functions and SA accumulation in germinating seeds, we examined the effects of exogenous SA (0–100 µM) on the germination of mutant and control seeds. The SA treatment inhibited the germination of the Col-0 WT seeds in a dosage-dependent manner (Figure 4). The germination of the *mes7* mutant seeds was delayed by SA, but not as much as the germination of the Col-0 WT seeds. Both of the *mes7* mutant and control seeds were relatively unaffected by the 0.1 μM SA treatment. The Col-0 WT seeds were sensitive to 1 μM SA, whereas the *mes7* mutant seeds were sensitive to 10 μM SA. Following the 10 μM SA treatment, 87.11% (*mes7-1*) and 88.82% (*mes7-2*) of the seeds from the two *mes7* mutants germinated to produce fully expanded green cotyledons. In contrast, the greening rate of Col-0 WT seeds was only 40.32% (Figure 4A,C). The application of an excessive amount of SA (100 μM) similarly inhibited the germination of the *mes7* mutant and Col-0 WT seeds (Figure 4B,C), which implied that the high exogenous SA concentration (100 µM) substantially exceeded the difference of the endogenous SA content between the *mes7* mutants and the Col-0 WT. MES7 displayed SA-dependent regulatory effects on seed germination under normal conditions, which further support our hypothesis that MES7 regulates seed germination via its activity in SA regulation.

### 2.5. MES7 SA-Dependently Facilitates Seed Germination under Saline Condition

**Figure 5:**
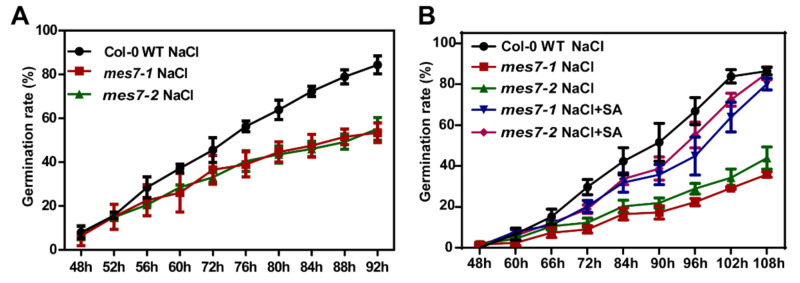
Application of exogenous SA rescues the germination deficiency of the *mes7* mutants under saline conditions. (**A**) Time course quantification of germination on 1/2 MS medium containing 150 mM NaCl. (**B**) Effects of low concentrations (10 μM) of SA on germination of *mes7* mutant seeds in the presence of 150 mM NaCl. Data from three independent replicates are shown and each biological repeat contains about 50 seeds. Error bars indicate ± SEM of the mean.

Previously study showed that low concentrations of SA facilitated seed germination under high salinity [14]. Moreover, salt stress has a more significant suppressive effect on SA-deficient plants than that on Col-0 WT plants [31]. To further elucidate the role of MES7 on seed germination, we evaluated the MES7 regulatory effects on seed germination upon a salt stress treatment. We carried out germination assays using *mes7* mutant seeds in the presence of 150 mM NaCl. Compared with the results in Figure 3A,B, the germination of both Col-0 WT and *mes7* mutants were significantly delayed by 150 mM NaCl, and the inhibition of *mes7* mutants were more serious (Figure 5A). The application of 10 µM exogenous SA rescued the germination deficiency of the *mes7* mutants and mitigated the inhibitory effect of the salt stress conditions on seed germination (Figure 5B). Taken together, we concluded that the regulation of *MES7* on seed germination is SA-dependent.

## 3. Discussion

In this study, we found that the malfunction of *MES7* promotes and delays the seed germination under normal or salt stressed condition, respectively. The accumulation of SA was decreased in the *mes7* seedlings, and the application of exogenous SA could rescue the germination deficient phenotype of *mes7*. These results indicate that *MES7* is a key regulator that modulates the seed germination under different conditions.

Seed germination is one of the major developmental phase transitions that are substantially affected by environmental stresses. This process is tightly regulated by hormone signaling pathways. The different effects of SA on seed germination were attributed to the growth status of seeds. Under normal conditions, SA increases reactive oxygen species (ROS)-mediated oxidative damage and induces H_2_O_2_ production, which adversely affects germination [32,33]. The application of exogenous SA inhibits seed germination under normal growth conditions [14,15,16]. Consistent with these earlier investigations, we observed that an application of exogenous SA inhibits seed germination under normal conditions (Figure 4B). In addition to affecting germination under normal condition, SA also increases the resistance to various abiotic stresses, minimizes ROS-mediated oxidative damage, and enhances seed germination followed by an exposure to stress [34,35]. In our study, decreasing the content of SA by mutating MES7 reduced the germination rate under salinity condition, which also proved the role of SA in plant resistance to abiotic stress (Figure 5).

The accumulation of SA is tightly controlled by multiple pathways related to SA synthesis and storage [20,36]. Although the SA functions related to seed germination have been investigated, how synthesis- or storage-related pathway modulates seed germination remains unknown. The salicylic acid induction deficient 2 (SID2) enzyme is essential for the SA synthesis pathway, with a mutation to SID2 leading to a significant decrease in the endogenous SA level [37]. However, SID2 does not affect seed germination under normal conditions [31]. The *NahG* gene encodes an SA hydroxylase, which degrades SA to catechol [38]. The overexpression of *NahG* decreases SA content, but does not change the seed germination under normal conditions. In this study, the accumulation of *MES7*, which encodes an enzyme that hydrolyzes MeSA to produce SA, decreases significantly in imbibed seeds. The malfunction of *MES7* promotes and inhibits seed germination under normal and saline conditions, respectively. Moreover, SA accumulation decreased in *mes7* seedlings, and the application of exogenous SA rescues the germination deficiency of the mutant seeds. These results suggested that the MeSA-to-SA conversion is important in the processing of germination (Figure 1A and Figure 5A).

The *MES* gene family members encode enzymes that hydrolyze MeSA, MeJA, or MeIAA in plants [26]. The functions vary among the MESs. Specifically, MES1, MES2, MES3, MES7, MES9, MES16, MES17, and MES18 can mediate the hydrolysis of MeIAA. In contrast, MES1, MES2, MES4, MES7, and MES9 hydrolyze MeSA, whereas MES1, MES2, MES3, MES9, MES10, and MES16 can hydrolyze MeJA [26,27,28]. However, which MES mediates the MeSA-to-SA conversion during seed germination has not been determined. We here revealed that the malfunction of *MES7* results in a decrease in SA content, and subsequently promotes seed germination under normal growth condition (Figure 4A,B). Moreover, an exogenous SA application rescues the germination difference of *mes7* mutants. Therefore, MES7 may play an important role in the MeSA-to-SA conversion and is crucial to the SA-mediated regulation of seed germination.

## 4. Materials and Methods

### 4.1. Plant Material and Growth Conditions

*A*. *thaliana* plants were grown at 22 °C under 12 h light/12 h dark conditions in a greenhouse. For germination assays, seeds were sown in a petri dish containing 1/2 MS medium (150 mM NaCl and/or different concentrations of SA was added according to the experimental requirements), vernalized at 4 °C for 3 days under dark conditions and then grown in an incubator at 22 °C under 12 h light/12 h dark conditions. The germination time was calculated from the time when the petri dish was put into the incubator, and the germination rate of seeds was counted at intervals.

### 4.2. Identification of T-DNA Insertions

Seeds of the *MES7* mutant lines (Salk_051188c and Salk_054303c) [27] were obtained from Arabidopsis Biological Resource Center. The T-DNA insertion salk line mutants *mes7-1* (Salk_051188c) and *mes7-2* (Salk_054303c) were verified by PCR amplification as previously described [39]. Three different primers were used for genotyping. LB was a T-DNA specific primer while LP and RP were designed according to the genomic DNA sequences. LB and RP were used to determine the T-DNA insertions while LP and RP were used to amplify the DNA fragments that containing T-DNA insertions. Primer sequences were shown in Supplemental Table S1.

### 4.3. RNA Extraction

The extraction of RNA from *A. thaliana* seeds was performed as described [40]. Briefly, RNA was extracted from seeds by an RNA extraction buffer (EB) with high pH. The buffer contains 100 mM Tris-HCl (pH 9.5), 5 mM DTT, and 1% sarkosyl to remove the polysaccharides and polyphenol. 50–100 mg dry seeds were grounded with liquid nitrogen into a fine powder. 1.2 mL EB was added before the sample thawed. After mixing, the supernatant was obtained by centrifuge at 11,000× *g* for 5 min. Then 0.5 volume of chloroform and acid phenol were added successively and the mixtures were centrifuged at 11,000× *g* for 15 min. The upper aqueous phase was carefully removed, and RNA was precipitated with 90 μL 3 M sodium acetate (pH 5.2) and 600 μL isopropanol. After −20 °C incubation 4 h and 10 min 11,000× *g* centrifugation, the upper aqueous phase was removed and the pellet was washed with 1 mL 75% ethanol (*v*/*v*). The pellet was resuspended in 1 mL TRIzol Reagent (Invitrogen, Carlsbad, CA, USA, 15596026) to extract RNA. The purified RNA was obtained by precipitation with isopropanol and washed with 75% ethanol (*v*/*v*).

Total RNA was extracted from 4-week-old plants using the TRIzol Reagent according to manufacturer’s instructions and a previous study [41]. Briefly, 0.1 g plant samples (other than seed, such as root, stem, flower, rosette leaf, etc.) were grounded in liquid nitrogen and mixed with 1 mL TRIzol Reagent for RNA isolation. RNA was precipitated with 2.2-fold volume absolute ethanol at −20 °C overnight. The precipitates were collected by centrifugation for 15 min 12,000× *g* at 4 °C and washed with 75% ethanol (*v*/*v*). The purified RNA was obtained by dissolving the precipitate with RNase-free water.

### 4.4. Reverse Transcription and qRT–PCR

To determine the expression levels of *MES7*, mRNA reverse transcription was performed after total RNA extraction. 1 μg total RNA was used for reverse transcription using PrimeScript ™ RT reagent Kit with gDNA Eraser (Takara, Wan Chai, HongKong, RR047A). The qRT-PCR was performed on a step one plus Real-Time PCR™ System (Applied Biosystems, Foster City, CA, USA) using TB Green™ *Premix Ex Taq*™ kit (Tli RNase H Plus) (Takara, RR820A). The final volume of 10 μL contained 5 μL 2 × TB Green Premix Ex Taq, 2 μL cDNA template (diluted into 1/10-fold before use), 200 nM of primers and ddH_2_O. The PCR profile was as follows: 95 °C for 30 s; 40 cycles of 95 °C for 5 s and 60 °C for 30 s. In order to identify a suitable internal control for qRT-PCR analysis during seed germination, the M values of eight housekeeping genes, *ACTIN2,* elongation factor 1α (*EF1α*)*,* polyubiquitin 10 (*UBQ10*)*,* adenine phosphoribosyl transferase 1 (*APT1*)*,* tubulin 6 (*TUB6*)*,* tubulin 8 (*TUB8*)*,* polyubiquitin 5 (*UBQ5*)*,* glyceraldehyde-3-phosphate dehydrogenase (*GAPDH*), were detected. *GAPDH* was finally selected as internal control for seed. Regarding to qRT-PCR for other samples, *ACTIN2* was used as endogenous control. The qRT-PCR analysis consisted of three technical repeats and three biological repeats. The primers were listed in Supplemental Table S1. The relative fold change in expression level was calculated using the 2^−ΔΔCt^ method.

### 4.5. Subcellular Localization

To determine the subcellular localization of MES7, *MES7* protein coding domain was amplified from cDNA and cloned into pENTR constructs using pENTR/SD/D-TOPO cloning kit (Invitrogen, K242020) according to the instruction of the manufacture. pEarlyGate102 (pEG102)-*MES7* was generated by LR reaction with pENTR-*MES7* [42]. The primers used for cloning were listed in Supplemental Table S1. Nucleus marker NLS-mCherry [43] constructs and pEG102-*MES7* (*p35s::MES7-CFP*) were transformed into *Agrobacterium tumefaciens* GV3101, and infiltrated into *Nicotiana benthamiana* at OD_600_ = 1.2.

Fluorescence microscopy analyses were performed at 72 h post inoculation (hpi) with Zeiss LSM-710 confocal microscope (Carl Zeiss, Thornwood, NY, USA). Cyan fluorescent protein (CFP) fluorescence and red fluorescent protein (RFP) fluorescence were excited under 505 nm and 543 nm, and visualized under 470–530 nm and 620–630 nm, respectively. Zen Black software was used to collect and process data.

### 4.6. Analysis of SA and IAA Accumulation

SA extraction and detection were performed as described previously [44,45]. Briefly, more than three sets of *A. thaliana* seedlings with different germination time were collected for each sample to detect the SA and IAA contents. 150 mg of each sample was collected, and grounded into fine powder in liquid nitrogen. 1 mL ethyl acetate spiked with 200 ng of D_4_-SA or D_5_-IAA was added to each sample and used as the internal standards for SA or IAA. After 10 min 11,000× g centrifugation at 4 °C, supernatants were evaporated and resuspended in 0.5 mL 70% methanol (*v*/*v*). The mixers were then centrifuged to clarify phases.

The supernatants were pipetted into glass vials and analyzed by HPLC-MS/MS (LCMS-8040, Shimadzu, Kyoto, Japan). Measurements were conducted on a LC-20AD liquid chromatography system (Shimadzu). At a flow rate of 0.3 mL/min, 10 μL sample was injected onto a ODS column (1.6 μm, 75 × 2 mm) (Shim-pack XR-ODS III). A mobile phase composed of solvent A (0.05% formic acid, 5 mM ammonium formate) and solvent B (methanol) was used in a gradient mode for the separation. SA or IAA was quantified by comparing its peak area with the peak area of its respective internal standard.

### 4.7. Statistical Analysis

Statistical analysis of germination: three biological repeats were performed for each assay and 30~50 seeds were used for each repeat. The seed germination rate was evaluated by counting the percentage of seeds with white radicles exposed, and the greening rate was assessed by counting the percentage of seeds with fully expanded green cotyledons. The seed germination rates were then calculated at each time point.

Statistical analysis was performed with one-way analysis of variance with post hoc Tukey honestly significant difference test [46]. According to Tukey’s test different lowercase letters indicate significant differences between multiple samples. According to the student *t*-test, asterisks mark significant differences between two treatments or two samples, * *p* < 0.05; ** *p* < 0.01; *** *p <* 0.001; **** *p <* 0.0001, and ns indicates that the difference is not significant.

Analysis of reference genes: M values of eight housekeeping genes were calculated by NormFinder software.

## 5. Conclusions

MES7 reduces seed germination through a SA-dependent manner under normal conditions and promotes seed germination under saline condition. Furthermore, the dysfunction of *MES7* decreases endogenous SA content. The application of SA restores the seed germination deficiencies of *mes7* mutants under different conditions. Taken together, our observations indicate that *MES7*, a MeSA hydrolytic enzyme, decreases SA accumulation, and SA-dependently modulates seed germination under normal and salt stress conditions. Our results have broad implications for clarifying the molecular basis of the regulation of seed germination.

## Data Availability

The data presented in this study are available in article and supplementary material.

## Supplementary Materials

The following are available online at https://www.mdpi.com/article/10.3390/plants10050903/s1, Table S1: Oligos used in this study.
