# Supplementary material for: MES7 Modulates Seed Germination via Regulating Salicylic Acid Content in Arabidopsis
Source: Plants (Basel). 2021 Apr 30;10(5):903. doi: 10.3390/plants10050903 (PMC8146826; doi:10.3390/plants10050903)
Supplement: Supplementary file 1 [file plants-10-00903-s001.zip › plants-1170995-supplementary.pdf]

Supplemental Table 1. Oligos used in this study.

Primer name	Sequence (5′-3′)
SALK_051188c LP	TCGTAAACATAAGCAGGTGGG
SALK_051188c RP	TTTTGAATTGGCGAAAATGTC
SALK_054303c LP	GAAGCAAATAGCATGAGCACC
SALK_054303c RP	TTATGTCGGGCATAAAAGCTG
LBb1.3	ATTTTGCCGATTTCGGAAC
MES1-qRTF	AGCTGCCTCCGGAATAGACA
MES1-qRTR	TTGGCAATGAGGTCAGGAGC
MES2-qRTF	GCTTTGGGCCATCGTGTAAC
MES2-qRTR	TCGACGAACGATGGTGAGTG
MES4-qRTF	TGGTGAGAGTAAACCCCATCAT
MES4-qRTR	GTACCGCCATGTCCTCTCCA
MES7-qRTF	CGCCTGCTTACGTTTTCCAA
MES7-qRTR	TGTCATGAACGGGCTTACCC
MES9-qRTF	TGGACTTCGAGTTAGGGAGC
MES9-qRTR	GCCGGTGTGACTCTCATCAA
ACTIN2-qRTF	AGTGGTCGTACAACCGGTATT
ACTIN2-qRTR	GATGGCATGAGGAAGAGAGAA
MES7-cdsF	CACCATGGATAAGAATAACCAGAAGAAGT
MES7-cdsR	GGCGTATTTATCTGCAATCTCCAAG
EF1α-qRTF	GATTTGCTGTTGTAACAAGATGGATG
EF1α-qRTR	AGGGTTGTATCCGACCTTCTTCA
UBQ10-qRTF	AGATCCAGGACAAGGAAGGTATTC

UBQ10-qRTR	CGCAGGACCAAGTGAAGAGTAG
APT1-qRTF	GTTGCAGGTGTTGAAGCTAGAGGT
APT1-qRTR	TGGCACCAATAGCCAACGCAATAG
TUB6-qRTF	CCACTGGTCGTTACGTTGGA
TUB6-qRTR	TGAGAATTGCACGGGGAACA
TUB8-qRTF	ACAGCCTCAGCCATGTTCAG
TUB8-qRTR	CGATGTCGAGTTCCCGATGA
UBQ5-qRTF	GTAAACGTAGGTGAGTCCA
UBQ5-qRTR	GACGCTTCATCTCGTCC
GAPDH-qRTF	TCTGACCTTGACATTGTTTCCA
GAPDH-qRTR	TCTCCAGTCCTTCATTGATGG
